# Supplementary material for: A Ruthenium(II) N-Heterocyclic Carbene (NHC) Complex with Naphthalimide Ligand Triggers Apoptosis in Colorectal Cancer Cells via Activating the ROS-p38 MAPK Pathway
Source: Int J Mol Sci. 2018 Dec 9;19(12):3964. doi: 10.3390/ijms19123964 (PMC6320930; doi:10.3390/ijms19123964)
Supplement: Supplementary file 1 [file ijms-19-03964-s001.pdf]

Supplementary Material

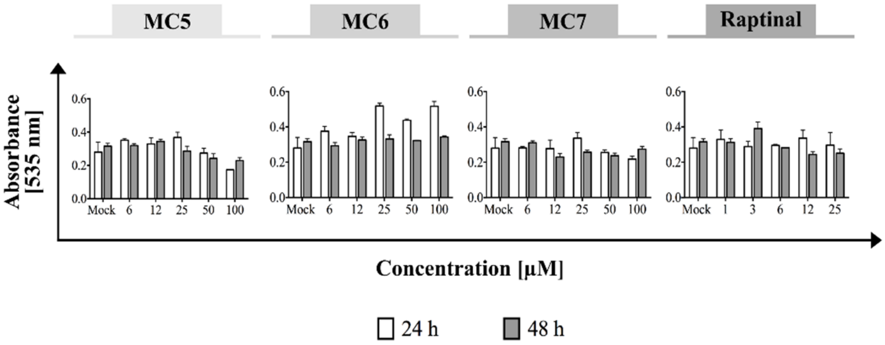

Figure S1. Human fibroblasts are less susceptible to the cytotoxic effects of naphthalimide-NHC analogues. HFF cells were treated with various concentrations of the respective compound as well as the rapid apoptosis inducer, raptinal [16] (as reference) for 24 and 48 h, and cytotoxicity was evaluated by SRB assay. 0.1% DMSO was used as mock treatment. Data are presented as mean ± SD of three independent experiments, each was done in quadruplicates.

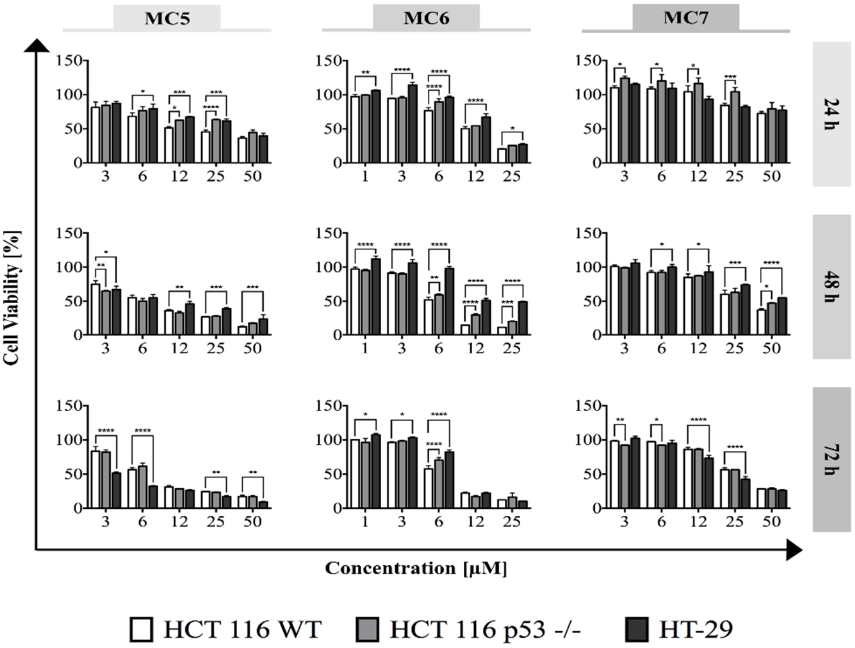

Figure S2. Cytotoxic efficacy of naphthalimide-NHC analogues in CRC cells harboring deficient- or mutant p53. Colon carcinoma cells representing three different p53 variations; HCT116 WT, HCT116 p53-/- and HT-29 (mutant; R273H) were treated with increasing concentrations of the respective compound for the indicated time points, thereafter evaluated by SRB cytotoxicity assay. Multiple comparisons were employed using two-way ANOVA test and a post-hoc Tukey test. *, **, ***, and **** represent p-values less than or equal to 0.05, 0.01, 0.001, and 0.001, respectively.

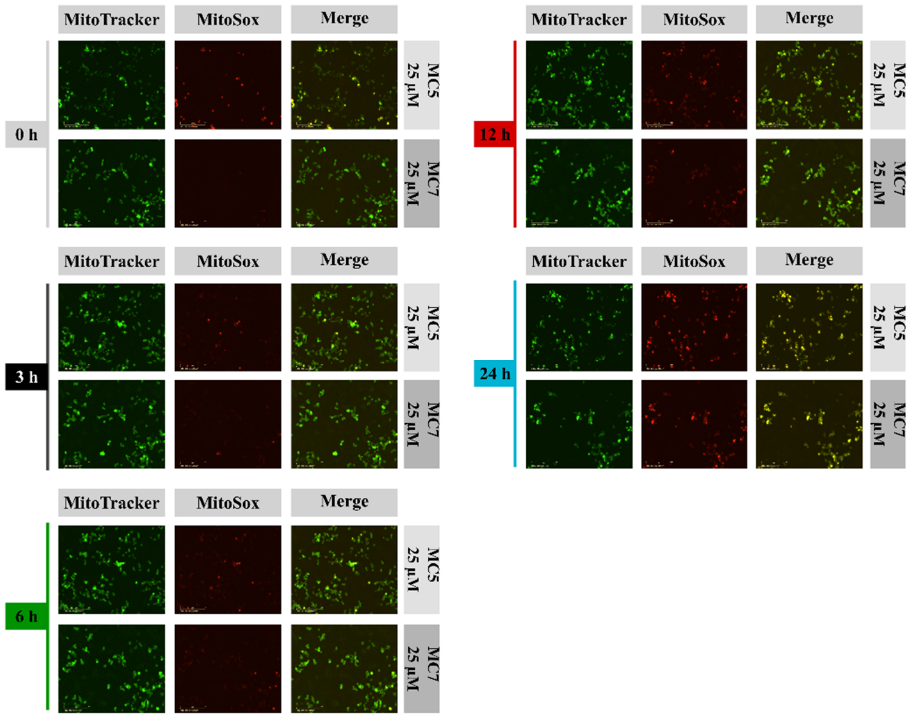

Figure S3. MtROS is strongly influenced by naphthalimide-NHC analogues. HCT116 cells were treated with the indicated concentrations of the compounds and the mitochondrial superoxide indicator, MitoSox Red, after which live cell imaging was performed every 30 mins for a period of 24 h. MitoTracker Green was used to indicate mitochondria.

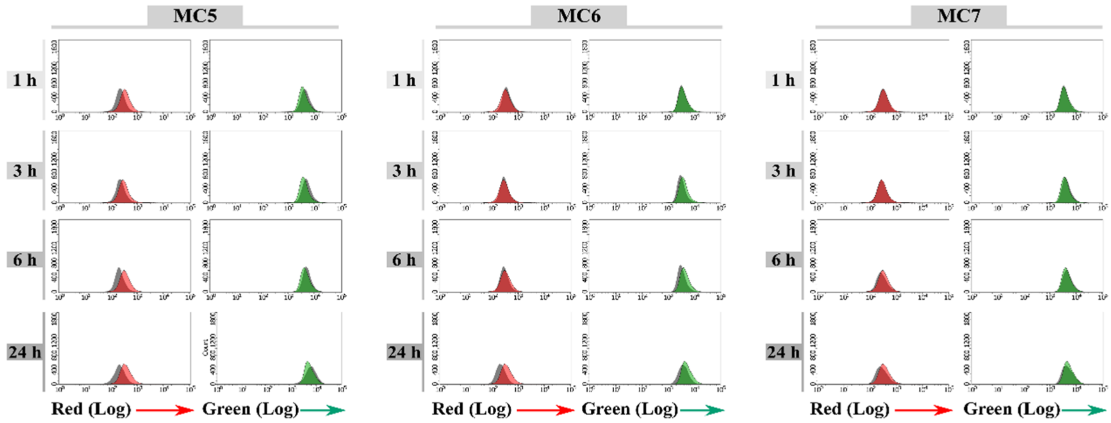

Figure S4. Naphthalimide-NHC analogues reduce, induce and have no clear effect on the MMP ($\Delta\psi_m$) in HCT116 cells. Representative histograms of flow cytometric analysis of MMP after treatment with MC5 (25 µM), MC6 (12 µM) and MC7 (25 µM) for the indicated time points. Dark (either red or green) curve, mock; light curve, treated cells. A rightward shift (increase) in green fluorescence indicates mitochondrial swelling and a leftward shift (decrease) in red fluorescence determines loss of $\Delta\psi_m$.

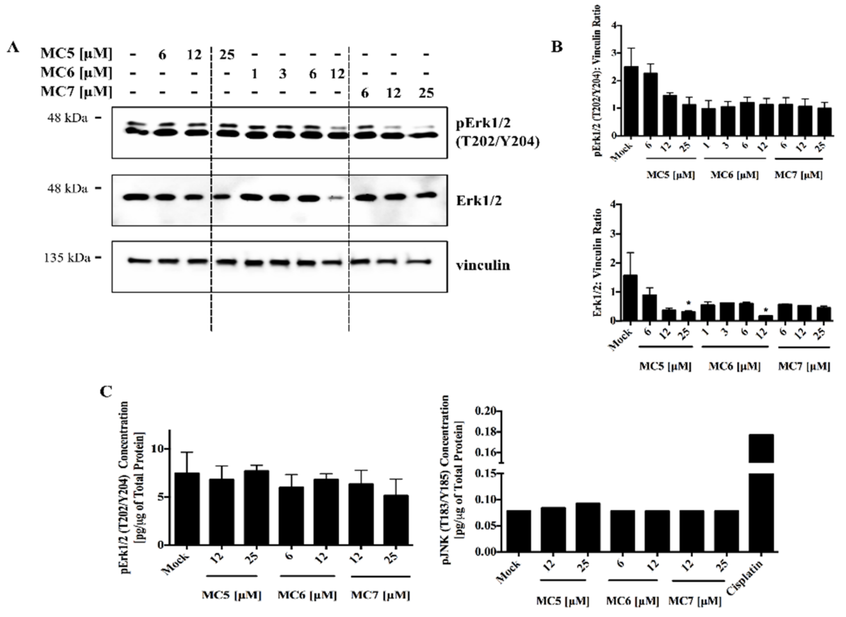

Figure S5. Naphthalimide-NHC analogues do not appear to profoundly impact the activation of ERK and JNK MAPKs. (**A**) HCT116 cells were treated with increasing concentrations of the three compounds for 24 h, as indicated, thereafter were subjected to immunoblotting for the detection of phospho- and total levels of Erk1/2; (**B**) Densitometric quantification of the presented bands in (**A**) demonstrate a modest reduction of both total Erk1/2 as well as its active form, pErk1/2 (T202/Y204). Error bars ± SEM, $n = 3$; (**C**) Phosphorylation status of Erk1/2 and JNK was determined by ELISA-microarray analysis after 24 h treatment with the three analogues at the indicated concentrations. Cisplatin (50 µM, 24 h) was used as positive control [11]. Statistical comparison was made between mock (0.1% DMSO) and the respective treatment using two tailed student's *t*-test. A *p*-value less than or equal to 0.05 is denoted as *.

© 2018 by the authors. Licensee MDPI, Basel, Switzerland. This article is an open access article distributed under the terms and conditions of the Creative Commons Attribution (CC BY) license (http://creativecommons.org/licenses/by/4.0/).
